# Mechanical, Crystallization, Rheological, and Supercritical CO_2_ Foaming Properties of Polybutylene Succinate Nanocomposites: Impact of Carbon Nanofiber Content

**DOI:** 10.3390/polym16010028

**Published:** 2023-12-20

**Authors:** Zhou Chen, Xichen Yin, Hui Chen, Xuguang Fu, Yuyue Sun, Qian Chen, Weidong Liu, Xiao Shen

**Affiliations:** 1 School of Mechanical and Power Engineering, Nanjing Tech University, Nanjing 211800, China; 202261107032@njtech.edu.cn (X.Y.); 202221106004@njtech.edu.cn (Y.S.); 202261207123@njtech.edu.cn (Q.C.); 202161207113@njtech.edu.cn (W.L.); 202161207067@njtech.edu.cn (X.S.); 2 Jiangsu Zhongneng Polysilicon Technology Development Co., Ltd., Xuzhou 221000, China; liuhui@gcl-power.com (H.C.); fuxuguang@gcl-power.com (X.F.); 3 Wuhu Innovation New Materials Co., Ltd., Wuhu 241080, China

**Keywords:** polybutylene succinate (PBS), carbon nanofibers (CNFs), microcellular foams, electrical conductivity, EMI shielding

## Abstract

As a substitute for conventional polymers for the preparation of biodegradable microcellular polymeric foams, polybutylene succinate (PBS) presents one of the most promising alternatives. However, the low melt strength of PBS makes it difficult to produce high-performance microcellular foams. This study aimed to improve the melt strength of PBS and explore the mechanical, thermal, crystalline, rheological, and supercritical CO_2_ foaming properties of PBS nanocomposites by using carbon nanofibers (CNFs). This study found that nanocomposites containing 7 wt% CNF exhibited the highest tensile strength, Young’s modulus, and bending strength. Moreover, the CNF nanofillers were well dispersed in the PBS matrix without significant agglomeration, even at high filler concentrations. Furthermore, the nanocomposites demonstrated improved melting temperature and crystallinity compared with pure PBS. The rheological analysis showed that the addition of CNFs significantly increased PBS viscosity at low frequencies due to the interaction between the PBS molecular chains and CNFs and the entanglement of CNFs, resulting in a more complete physical network formation when the CNF content reached above 3 wt%. During the supercritical CO_2_ foaming process, the addition of CNFs resulted in increased cell density, smaller cells, and thicker cell walls, with good laps formed between the fibers on the cell walls of nanocomposite foams. Moreover, the electrical conductivity and electromagnetic interference (EMI) shielding properties of the foamed material were studied, and a nanocomposite foam containing 7 wt% CNF showed good electrical conductivity (4.5 × 10^−3^ S/m) and specific EMI shielding effectiveness (EMI SE) (34.7 dB/g·cm^−1^). Additionally, the nanocomposite foam with 7 wt% CNF also exhibited good compression properties (21.7 MPa). Overall, this work has successfully developed a high-performance, multifunctional PBS-based nanocomposite foam, making it suitable for applications in various fields.

## 1. Introduction

Polymeric foams are widely used in various industries due to their exceptional characteristics. Their lightweight nature makes them ideal for load-sensitive applications without compromising their strength. Additionally, their high specific strength ensures durability and resilience under stress. Moreover, these foams provide excellent heat and sound insulation, which is ideal for environments where thermal management and noise reduction are crucial. Owing to these versatile properties, polymeric foams are extensively used in fields like automotive and aerospace engineering for weight reduction, and in construction and packaging for insulation and cushioning. Traditional foam materials, including polystyrene (PS) [1,2], polyurethane (PU) [3,4], polyvinyl chloride (PVC) [5], and ethylene vinyl acetate (EVA) [6] are commonly used in the modern world. However, increasing plastic pollution has led to concerns regarding the risks associated with waste plastics and the difficulty in disposal of traditional polymeric foams due to their non-biodegradable nature [7]. In view of this, researchers are exploring the development of environmentally friendly and biodegradable polymeric foams to replace non-biodegradable ones [8,9,10,11,12,13,14,15,16,17]. Among these, polybutylene succinate (PBS) is a promising biodegradable plastic due to its good mechanical properties, processability, heat resistance, and biodegradability [18,19]. PBS foams are considered as a viable alternative to traditional polymeric foams [20,21,22]. However, the use of PBS materials in fabricating high-performance biodegradable foams faces intrinsic challenges. A primary issue is their low melt viscoelasticity and strength resulting from their low molecular weight. The narrow molecular weight distribution of PBS further exacerbates this issue. In particular, their inherent linear chain structures bring about these limitations. Overall, these factors complicate the processing and production of high-quality biodegradable PBS foams. Overcoming these challenges is crucial to enhance PBS’s practicality in industries prioritizing environmental sustainability and biodegradability.

In recent years, the supercritical fluid foaming method has garnered considerable interest due to its environmentally friendly, non-toxic, and straightforward process, making it an attractive option for producing high-performance PBS foams [23]. In the realm of supercritical fluid foaming technology, supercritical carbon dioxide (ScCO_2_) is commonly employed as the foaming agent. This choice is primarily due to its minimal environmental impact, which aligns with sustainable practices. The utilization of ScCO_2_ in this technology significantly mitigates the environmental footprint associated with polymer foam production. Researchers have made substantial strides in the past few decades in developing efficient methods for creating high-performance PBS foams using supercritical CO_2_ foaming [24]. Incorporating nanofillers into the PBS matrix has emerged as a simpler and more effective means of improving melt viscosity and strength, and thus enhancing its foaming behavior. For instance, Wu et al. reported that adding halloysite nanotubes to the PBS matrix resulted in a significant improvement in the nanocomposite’s complex viscosity, which contributed to better foaming performance [25]. A comparable outcome was observed in a previous study of the PBS/carbon black (CB) system [26]. By adding an appropriate amount of CB nanofiller to the PBS matrix, the nanocomposites crystallized faster, had higher thermal stability, and exhibited higher complex viscosities, all of which are beneficial during the supercritical CO_2_ foaming process. Recently, Wang et al. developed biodegradable PBS/carbon nanotube (CNT) foams with excellent electromagnetic interference (EMI) shielding properties by using a solid-state supercritical CO_2_ foaming method [27]. The CNTs served as reinforcing agents in the PBS melt, leading to a significant increase in the storage modulus and complex viscosity of the PBS matrix, thus improving the foaming properties of the resulting composite foams. They also observed that the incorporation of CNTs greatly improved the electrical and EMI shielding properties of the composite foams. Prior studies have indicated that adding nanofillers to the PBS matrix can improve both foaming properties and impart specific functionalities. In addition to CNTs, carbon nanofibers (CNFs) are another type of frequently used one-dimensional (1D) carbon nanofiller. CNFs share 1D nanostructures, special properties, and multifunctionalities with CNTs, as well as additional benefits such as lower costs, higher defect densities, and lower crystallinity [28,29,30]. To the best of our knowledge, there currently exists a gap in the scientific literature regarding the use of supercritical CO_2_ foaming technology for the fabrication of PBS/CNF nanocomposite foams.

The present investigation aims to fabricate high-performance polybutylene succinate/carbon nanofiber (PBS/CNF) nanocomposite foams through a combination of melt-compounding and supercritical CO_2_ foaming techniques. Initially, the PBS and CNF materials were blended via melt processing using a HAAKE torque rheometer. A comprehensive analysis was conducted on the fracture morphology, rheological behavior, crystallization, and mechanical performance of the PBS/CNF nanocomposites containing varying CNF nanofiller contents. Subsequently, the prepared PBS/CNF nanocomposites were foamed using supercritical CO_2_ as the blowing agent in a batch foaming procedure. The pore structure, pore characteristics, and compressive properties of the PBS/CNF nanocomposite foams were thoroughly examined. Additionally, the electrical and electromagnetic interference (EMI) shielding characteristics of the PBS/CNF nanocomposites and their nanocomposite foams were assessed. Overall, this study provides an approach for the fabrication of high-performance, PBS-based nanocomposite foams, presenting a significant advancement in the field of polymeric foams.

## 2. Materials and Methods

### 2.1. Materials

Poly (butylene succinate) (PBS) (TH801) was purchased from the Xinjiang Lanshan Tunhe Polyester Co., Ltd. (Changji, China). It has a density of 1.25 g/cm^3^ and a melt flow rate of 20 g/10 min at 190 °C/2.16 kg. Carbon nanofiber (CNF) (150–200 nm in diameter and 10–30 μm in length) was obtained from Beijing Deke Daojin Technology Co., Ltd. (Beijing, China). CO_2_, purified at 99.9% and was used as a physical blowing agent.

### 2.2. Preparation of PBS/CNF Nanocomposites and Their Nanocomposite Foams

**Figure 1:**
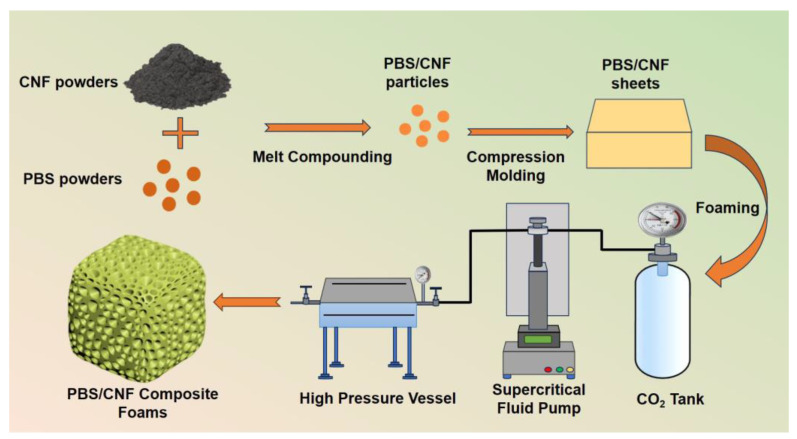
A schematic diagram illustrating the preparation process of the PBS/CNF composites and their nanocomposite foams.

As shown in Figure 1, the preparation process of foamed PBS/CNF nanocomposites involves several complex steps. Initially, both the PBS pellets and CNFs undergo a preparatory phase, which includes vacuum drying at 60 °C for 12 h. This step is crucial for removing any moisture that could affect the properties of the materials. Following this, a melt-blending process is employed. In this step, the dried PBS and CNF pellets are combined using a specific mixing instrument known as the HAAKE Polylab Open System. The blending occurs at a controlled temperature of 140 °C, and the mixture is stirred at a consistent speed of 60 rpm for a duration of 5 min. The precise control of temperature and stirring speed is vital to ensure a uniform blend of PBS and CNF. After blending, the next step involves compression molding of the samples. This is carried out at the same temperature as the blending process, 140 °C, but under a pressure of 5 MPa. The duration for this compression molding is also 5 min. The purpose of this step is to form the blended material into a specific shape and density, preparing it for the subsequent supercritical CO_2_ foaming process. The supercritical CO_2_ foaming procedure is well-documented in the scientific literature due to its effectiveness in creating foamed materials [31]. In this specific study, the supercritical CO_2_ foaming was conducted in a precisely controlled environment—a temperature of 110 °C and a pressure of 13.8 MPa. The saturation time for this process was set at 2 h. This step is pivotal as it introduces the supercritical CO_2_ into the PBS/CNF matrix, creating the foam structure within the nanocomposite. Finally, the resulting PBS/CNF nanocomposites and nanocomposite foams with varying CNF content were labeled as PBS*x* and F-PBS*x*, respectively (where *x* denotes the added mass fraction of CNF).

### 2.3. Characterization

#### 2.3.1. Scanning Electron Microscope (SEM) Test

To investigate the microscopic fracture morphology of PBS/CNF nanocomposites and their nanocomposite foams, we utilized scanning electron microscopy (SEM) imaging, employing a VEGA3 SBH-EasyProbe instrument (TESCAN, Brno, Czech Republic). Before SEM analysis, each sample was prepared through a two-step process: oven drying at 60 °C for 6h, followed by gold spraying for 3 min. Subsequently, the obtained SEM micrographs were analyzed with Image-Pro Plus 6.0 software (Media Cybernetics, Rockville, Matyland, MD, USA) to calculate both the average cell size and cell density of the samples.

#### 2.3.2. Rheological Properties Test

The rheological properties of PBS/CNF nanocomposites were evaluated using an MCR302 rotational rheometer (Anton Paar, Graz Austria). For measurement accuracy and consistency, all the samples were set to 25 mm in diameter and 1 mm in thickness. The tests were conducted at a temperature of 130 °C to observe viscoelastic behavior in high-temperature scenarios. The angular frequency range was set between 1 and 100 rad/s, enabling comprehensive analysis of the nanocomposites’ rheological responses.

#### 2.3.3. Mechanical Properties Test

PBS/CNF nanocomposites and their nanocomposite foams were tested using an Instron 5967 material testing machine (Instron, Boston, MA, USA). Tensile strength, Young’s modulus, and elongation at break of nanocomposites were obtained at a tensile rate of 5 mm/min. The bending strength and bending modulus of the nanocomposite materials were determined by three-point bending tests at a rate of 2 mm/min. The compression properties of nanocomposite foams were tested at a compression rate of 1 mm/min. A pendulum impact tester (SAN ZBC1000) was used at room temperature to determine the impact strength of the nanocomposite.

#### 2.3.4. Differential Scanning Calorimetry (DSC) Test

The DSC test was carried out using DSC 214 (NETZSCH, Selb, Germany). The nanocomposite was ramped up from room temperature to 140 °C at a rate of 10 °C /min under nitrogen protection; after 5 min of isotherm, the nanocomposite was cooled to 40 °C at the same rate. After an additional 5 min of isotherm, it was ramped up to 140 °C at a rate of 10 °C/min to obtain the secondary ramp curve. Equation (1) was used to determine the crystallinity of the PBS/CNF nanocomposites.

(1) $$\chi_{c}=\frac{{\Delta H}_{m}-{\Delta H}_{c}}{{\Delta H}_{m}^{0}}\times wt\%\times 100\%$$

where ${\Delta H}_{m}$ is the enthalpy of melting of the secondary heating curve, ${\Delta H}_{c}$ is the enthalpy of cold crystallization of the secondary heating curve, and ${\Delta H}_{m}^{0}$ is the enthalpy of melting of the fully crystallized PBS material.

#### 2.3.5. Electrical Conductivity Test

The electrical conductivity of the nanocomposites and nanocomposite foams was evaluated using different instruments based on the conductivity level. For samples exhibiting electrical conductivity higher than 10^−6^ S/cm, a four-point probe test instrument (RTS-9, Guangzhou, China) was employed. Conversely, for samples with electrical conductivity below 10^−6^ S/cm, a high-resistance meter (ZC36, Shanghai Anbiao Electronics Co., Ltd. Shanghai, China) was utilized. Each sample was tested five times to ensure accuracy and reliability, and the results were averaged to obtain the final conductivity value.

#### 2.3.6. Electromagnetic Interference Shielding Performance Test

The electromagnetic interference (EMI) shielding effectiveness (SE) of the PBS nanocomposites and their foam variants was assessed using a Keysight E5063A vector network analyzer in the X band (8.2–12.4 GHz). This frequency range is crucial for numerous wireless communication applications. Test samples, uniformly sized at approximately 22.86 mm in length, 10.16 mm in width, and 2.5 mm in thickness, were used in this analysis.

The calculations of various EMI SE, including the total shielding effectiveness (SE_T_), reflection shielding effectiveness (SE_R_), absorption shielding effectiveness (SE_A_), multiple shielding effectiveness (SE_M_), as well as the coefficients for absorption (A), transmission (T), and reflection (R) were performed as follows:

(2) $$R={\left|S_{11}\right|}^{2}={\left|S_{22}\right|}^{2}$$

(3) $$T={\left|S_{21}\right|}^{2}={\left|S_{12}\right|}^{2}$$

(4) A=1−R−T

(5) $${SE}_{R}=-10\mathrm{lg}(1-R)$$

(6) $${SE}_{A}=-10lg\left(\frac{T}{1-R}\right)$$

(7) $${SE}_{T}={SE}_{A}+{SE}_{R}+{SE}_{M}$$

The scattering parameters S_11_, S_12_, S_21_, and S_22_, which are integral to our measurements, represent the power of the output signal that is reflected back to port 1 (or 2) after being transmitted from port 1 (or 2) and subsequently reaching the receiver at port 2 (or 1). These parameters are crucial for understanding signal behavior in the system and are expressed in decibels (dB). Furthermore, it is important to note that the SE_M_ can generally be considered negligible when the SE_T_ exceeds 15 dB.

## 3. Results and Discussion

**Figure 2:**
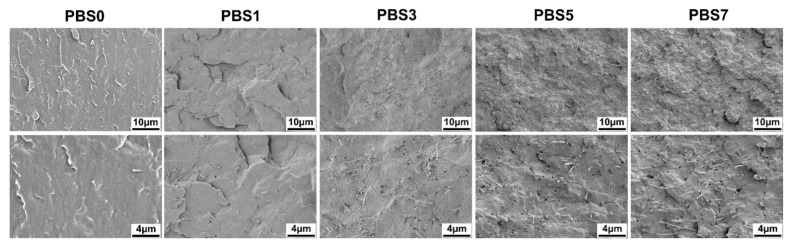
Fracture morphology of PBS/CNF nanocomposites with different nanofiller content.

Generally, uniform dispersion of nanofillers in a polymer matrix is essential for improving the performance of polymer composites. Homogeneous nanofiller distribution is vital as it affects the composite’s mechanical, thermal, and electrical properties. Effective dispersion optimizes nanofiller properties and enhances composite stability and durability [32,33]. Therefore, we initially analyzed the dispersion of CNF nanofiller in the PBS matrix using SEM. The morphology of brittle fracture surfaces in pure PBS and PBS/CNF nanocomposites is presented in Figure 2. As observed, the fracture surface of the pure PBS sample appeared smooth, while that of the composite exhibited apparent fiber breakage and partial pull-out. Notably, even with a CNF content as high as 7 wt%, no obvious agglomeration was observed in the PBS matrix. The dispersion of CNFs in the polymer matrix is crucial for improving the composite’s melt strength and foaming characteristics. However, it is worth mentioning that when the CNF content exceeds 3 wt%, visible fiber overlap can be observed in the figure. Successful construction of a conductive network can improve the composite’s conductivity, electromagnetic shielding efficiency, and pore homogeneity during foaming.

**Figure 3:**
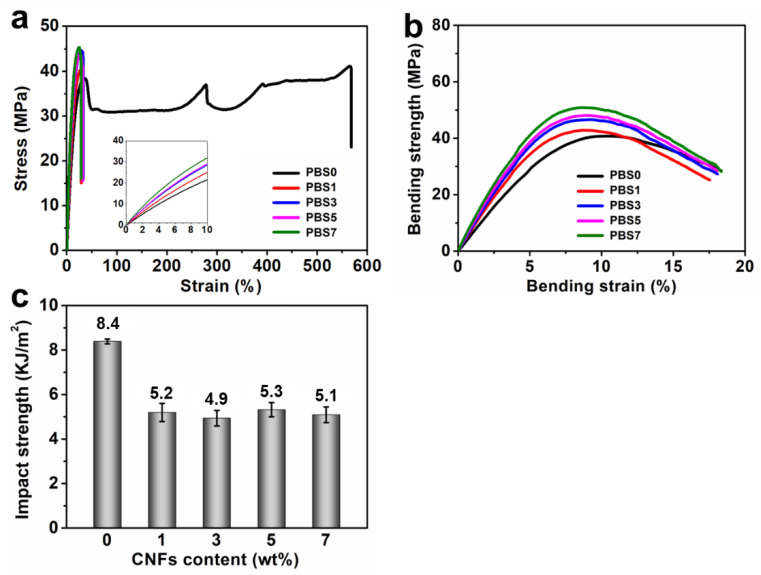
Mechanical properties of PBS/CNF nanocomposites: (**a**) tensile properties, (**b**) bending properties, and (**c**) impact toughness.

The present study investigated the influence of CNFs on the mechanical properties of PBS/CNF nanocomposites. The tensile, bending and impact properties of the nanocomposites were examined, and the results are presented in Figure 3 and Table 1. As demonstrated in Figure 3a, the addition of CNFs led to a significant improvement in the tensile strength and Young’s modulus of the nanocomposites. This improvement can be attributed to the reinforcing effect of the rigid CNFs. However, the incorporation of CNFs caused a notable reduction in the nanocomposites’ elongation at break when compared with pure PBS. The trend in the bending properties of the nanocomposites, as shown in Figure 3b, was consistent with their tensile properties and followed an increasing trend with increasing filler content. The toughness of the nanocomposites was assessed through impact tests. As evidenced in Figure 3c, the inclusion of CNFs decreased the toughness of the nanocomposites, which was consistent with the tensile results. It is important to note that the impact strength of a material reflects its toughness performance under sudden impact or slow stretching, whereas its elongation at break represents its ductility. Thus, the addition of CNFs to PBS increased the strength and modulus of the nanocomposites but led to a slight decrease in their toughness and ductility.

**Figure 4:**
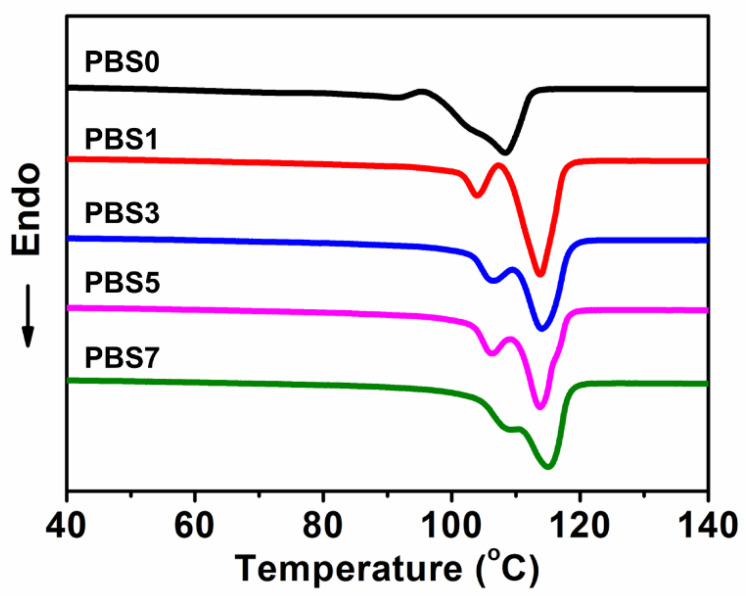
DSC second heating curves of PBS/CNF nanocomposites.

The DSC thermograms of the pure PBS and PBS/CNF nanocomposites are shown in Figure 4, while their corresponding melt temperatures and crystallinity results are presented in Table 2. The addition of CNF nanofillers to PBS significantly increased the crystallinity of the nanocomposites due to the heterogeneous nucleation effect of CNF nanofillers. With 7 wt% CNF added, the nanocomposites reached 33.5% crystallinity and 115.1 °C melt temperature, which are significantly higher than the values for pure PBS.

**Figure 5:**
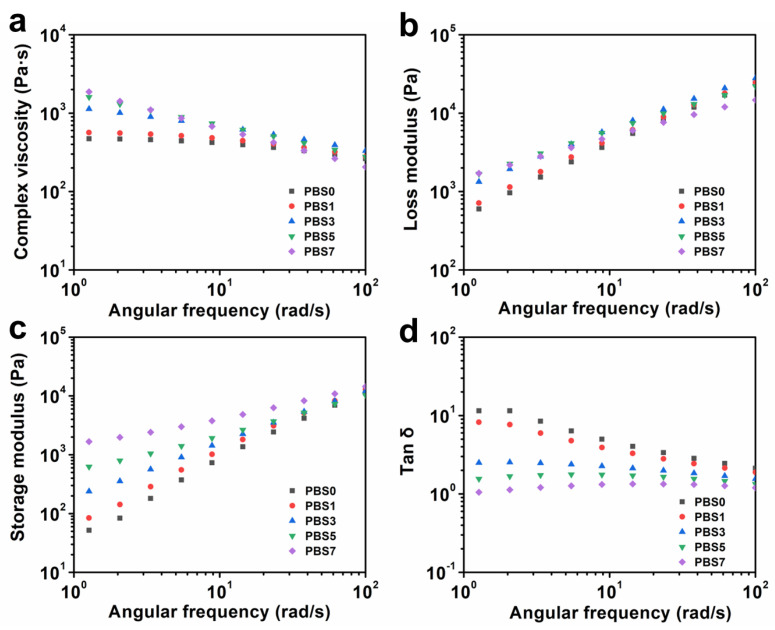
Rheological properties of PBS/CNF nanocomposites: (**a**) complex viscosity, (**b**) loss modulus, (**c**) storage modulus, and (**d**) tan δ.

The rheological behavior of PBS/CNF nanocomposites is illustrated in Figure 5, which shows that the complex viscosities of PBS/CNF nanocomposites are higher than those of pure PBS. This is attributed to the rigid CNF entanglement with PBS and increased entanglement between PBS molecular chains, resulting in a decrease in melt flow [34,35]. Moreover, a typical shear thinning phenomenon was observed in all samples across the entire frequency range. Furthermore, PBS/CNF nanocomposites exhibited higher modulus and loss modulus compared with pure PBS, which increased with increasing filler content but became less dependent on frequency as filler content increased (Figure 5b,c). Therefore, to form a more complete physical network, CNF content should be 3 wt% and above. As can be seen, CNFs can enhance the melt strength of PBS, which in turn, improves PBS’ foaming properties.

**Figure 6:**
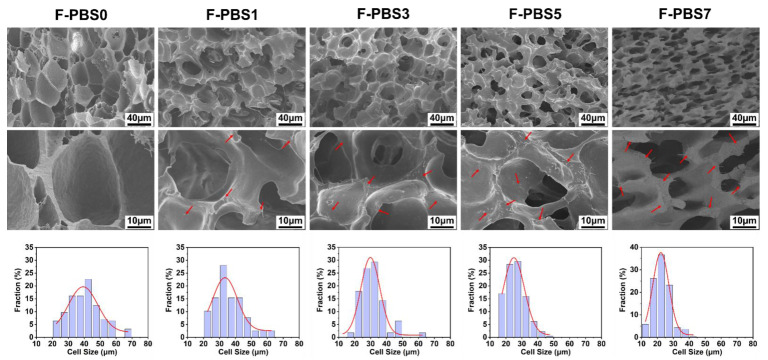
Cell morphology and cell size distribution of PBS/CNF nanocomposite foams with different CNF contents.

Figure 6 illustrates the cell morphology and cell size distribution of the PBS/CNF nanocomposite foams, while Table 3 presents the average cell size and density. All samples exhibit a uniform cell structure. As the CNF content increases, the cell size decreases, and cell density and cell wall thickness increase. This behavior can be attributed to the heterogeneous nucleation effect of CNF nanofillers, which results in more cell nuclei during the initial stages of cell formation. Moreover, the processes of cell nucleation and growth compete, leading to an increase in cell density and a decrease in cell size. Additionally, the rheological analysis revealed that the addition of rigid CNF nanofillers significantly enhances the melt strength of the nanocomposites, thereby preventing uncontrolled cell growth until rupture occurs. With the addition of 7 wt% CNF to PBS, the average cell size of the nanocomposite foams decreased by 50%, while the density increased from 1.8 × 10^7^ cells/cm^3^ to 1.3 × 10^8^ cells/cm^3^ relative to pure PBS foam. Remarkably, as the CNF content in the nanocomposite foams increased, the fibers retained their shape and were visible on the cell wall, thus positively impacting the foam’s conducting and EMI shielding properties.

**Figure 7:**
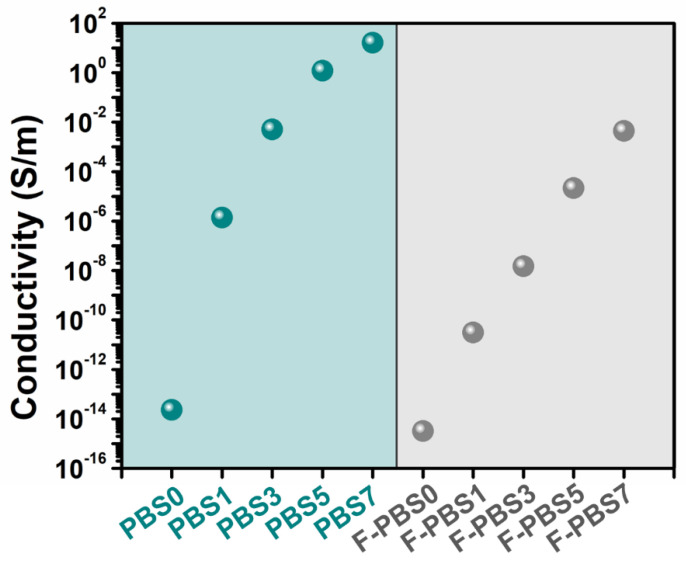
Electrical conductivity of PBS/CNF nanocomposites and their nanocomposite foams.

Figure 7 presents the electrical conductivity of the pure PBS and PBS/CNF nanocomposites, both before and after foaming. Pure PBS acts as an insulating material, whereas the nanocomposite conductivity increases with increasing CNF content, attributable to its high electrical conductivity. For instance, the electrical conductivity of the PBS7 sample was boosted by 15 orders of magnitude compared with pure PBS. Furthermore, the electrical conductivity of all the foamed samples was observed to decrease compared with their unfoamed counterparts. This can be attributed to uniform cell growth inside the composite during foaming, leading to an enlargement in CNF spacing. In addition, the stretching of the composite uniformly around the cells’ nuclei disrupted a part of the conductive network. Nevertheless, it is noteworthy that the F-PBS7 sample still maintained an electrical conductivity of 4.5 × 10^−3^ S/m, signifying that the CNFs in this foamed sample retained a relatively good conductive network.

**Figure 8:**
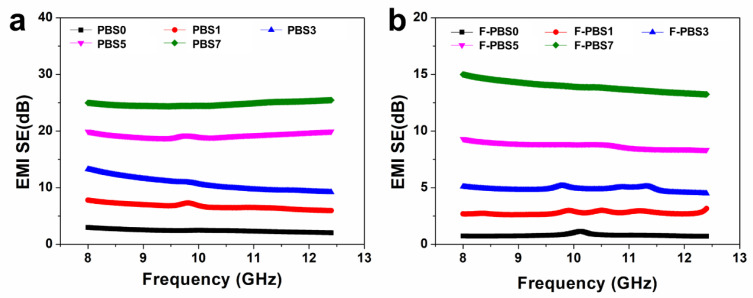
EMI shielding performance of PBS/CNF nanocomposites (**a**), and PBS/CNF nanocomposite foams (**b**).

**Figure 9:**
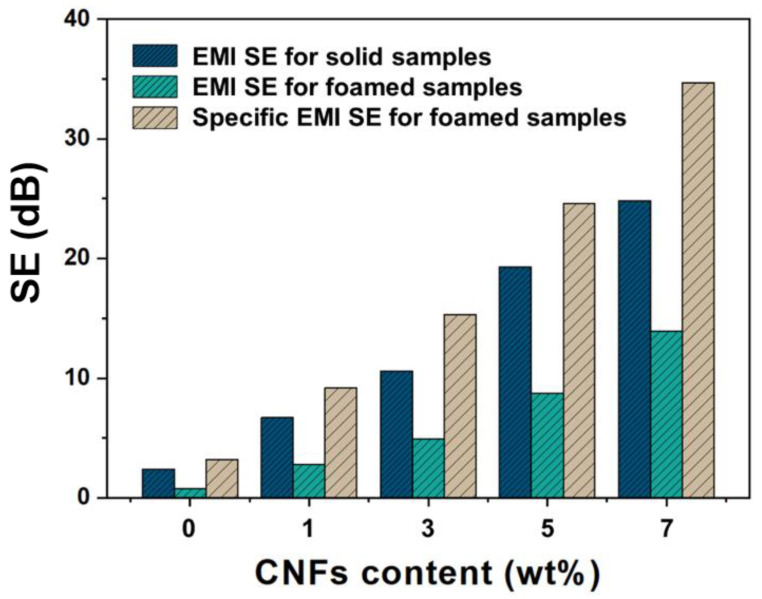
Specific EMI SE of solid and foamed samples.

The EMI shielding capabilities of polymeric nanocomposites are highly dependent on their electrical conductivity. In light of the electrical conductivity results previously discussed, an investigation into the EMI shielding performance of PBS/CNF nanocomposites and their corresponding nanocomposite foams was conducted. Figure 8a,b illustrate the EMI shielding effectiveness (SE) values of the nanocomposites and nanocomposite foams containing varying amounts of CNF nanofillers. It was observed that the EMI SE of both solids and foams was almost invariant to frequency, but closely related to the CNF nanofiller content. This is attributed to a high CNF content resulting in both greater electrical conductivity and electromagnetic shielding properties. For instance, at a CNF addition amount of 7 wt%, the average EMI SE of the PBS7 composites was found to be 24.8 dB, while that of F-PBS7 composite foams was 13.9 dB. Porous structures in foamed samples enable them to attain comparable EMI SE values to those of solid composites at lower CNF volume contents. Furthermore, specific EMI SE values are commonly employed to characterize the EMI shielding performance of foamed materials, and the specific EMI SE of foams reached up to 34.7 dB/g·cm^−1^ (Figure 9), surpassing that of solid samples. The incorporation of CNF nanofillers was shown to enhance the EMI shielding performance of biodegradable PBS-based composites and their composite foams.

**Figure 10:**
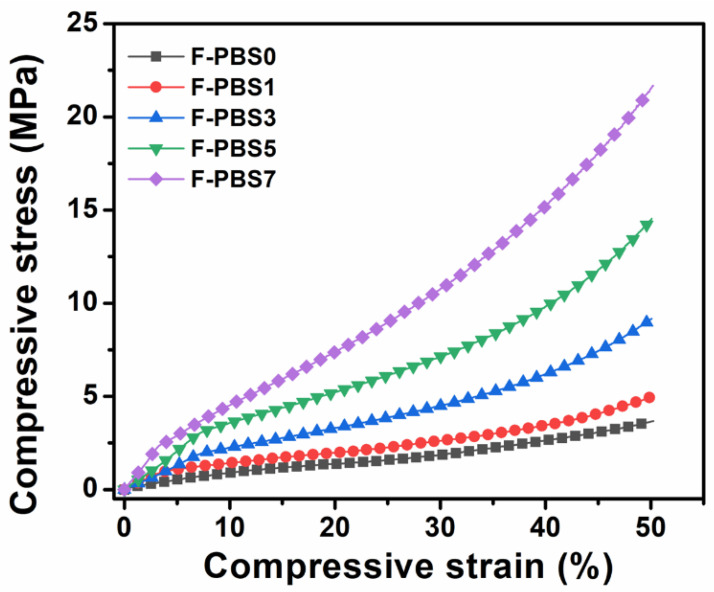
Compressive stress–strain curves of PBS/CNF nanocomposite foams.

Figure 10 presents results on the compressive properties of PBS/CNF nanocomposite foams, highlighting the impact of CNFs on their mechanical properties. Pure PBS foam, without the addition of CNFs, showed weak compressive strength, measuring just 3.7 MPa at 50% compression. This low performance indicates limited applications in scenarios requiring high mechanical strength. Incorporating rigid CNFs substantially improved the PBS foam’s compressive behavior. The results indicate a direct correlation between CNF concentration and the foam’s compressive strength. At 50% compression strain, PBS/CNF nanocomposite foam reached 21.7 MPa in compressive strength, markedly higher than the 5.0 MPa of F-PBS0, another PBS foam variant. SEM images reveal that CNF nanofillers form a reinforced structure within the foam’s cell walls, akin to concrete reinforcement. This reinforcement is crucial for understanding the enhanced compressive properties of CNF-reinforced PBS foams. In summary, these findings highlight CNFs’ potential to significantly boost PBS foams’ mechanical properties, expanding their application in areas demanding strength and durability. This material science advancement not only expands the uses of PBS foams but also underscores the role of nanocomposites in improving material properties.

## 4. Conclusions

In this work, we successfully developed lightweight, high-strength PBS/CNF nanocomposite foams using a combination technique of melt compounding and supercritical CO_2_ foaming. Incorporating CNFs into the PBS matrix significantly enhanced the mechanical and crystalline properties of the nanocomposites. Quantitatively, this enhancement led to significant improvements in the mechanical properties of the material. Specifically, the tensile strength increased to 45.3 MPa, representing an 11.0% enhancement, and the bending strength rose to 50.9 MPa, a 23.8% increase compared with pure PBS. Additionally, the crystallinity of the material also increased significantly, reaching 33.5%, which is a 38.4% increment over pure PBS. CNF nanofillers were effectively dispersed in the PBS matrix, preventing excessive agglomeration and increasing the nanocomposites’ complex viscosity. The use of supercritical CO_2_ foaming technology produced a uniform cellular structure in the nanocomposite foams. This resulted in reduced cell size and increased cell density, due to the heterogeneous nucleation by CNFs and increased melt strength of the nanocomposites. Additionally, the foams showed impressive EMI shielding properties, which were enhanced by the foams’ cellular structure. However, their electrical conductivity was relatively lower than that of the nanocomposites. Furthermore, the foams had commendable compressive strength, up to 21.7 MPa at 7% CNF content. Overall, this research presents a promising method for creating high-performance, multifunctional PBS-based nanocomposite foams suitable for various applications requiring lightweight and high-strength materials.

## Figures and Tables

**Table 1:** Mechanical properties of PBS/CNF nanocomposites.

Sample	Tensile Strength (MPa)	Young’s Modulus (MPa)	Elongation at Break (%)	Bending Strength (MPa)	Bending Modulus (MPa)	Impact Strength (KJ/m^2^)
PBS0	40.8 ± 0.4	250.3 ± 8.3	559.2 ± 12.6	41.1 ± 0.4	642.1 ± 3.2	8.4 ± 0.1
PBS1	41.1 ± 1.1	326.2 ± 29.1	27.1 ± 2.5	45.1 ± 1.2	770.4 ± 28.9	5.1 ± 0.4
PBS3	42.4 ± 1.2	372.2 ± 11.4	32.5 ± 0.9	46.3 ± 0.6	834.1 ± 23.4	5.2 ± 0.3
PBS5	43.6 ± 0.3	381.1 ± 12.9	31.0 ± 3.1	48.5 ± 0.5	922.7 ± 13.8	5.3 ± 0.3
PBS7	45.3 ± 1.5	417.5 ± 20.1	28.3 ± 0.4	50.9 ± 1.2	987.6 ± 17.7	5.1 ± 0.4

**Table 2:** Melting temperature, melting enthalpy and crystallinity of PBS/CNF nanocomposites.

Samples	T_m_ (°C)	∆H_m_ (J/g)	*X*_c_ (%)
PBS0	108.4	48.3	24.2
PBS1	113.8	58.3	29.2
PBS3	114.1	62.1	31.1
PBS5	113.8	65.2	32.6
PBS7	115.1	66.9	33.5

**Table 3:** Average cell size and cell density of nanocomposite foams.

	F-PBS0	F-PBS1	F-PBS3	F-PBS5	F-PBS7
Average cell size (μm)	41.0 ± 11.1	35.1 ± 8.9	31.3 ± 7.6	26.9 ± 4.2	22.0 ± 4.5
Average cell density (cells/cm^3^)	1.8 × 10^7^	3.2 × 10^7^	4.2 × 10^7^	7.3 × 10^7^	1.3 × 10^8^

## Data Availability

The data presented in this study are available on request from the corresponding author.
